# Rhamnolipid-modified biochar improves the microenvironment of saline soils to enhance soil productivity

**DOI:** 10.3389/fpls.2026.1721869

**Published:** 2026-03-09

**Authors:** Lingkun Jing, Lei Zhang, Yakang Liang, Muladili Abulaiti, Ziyi Zang, Hongbo Wang, Xingpeng Wang

**Affiliations:** 1 Key Laboratory of Comprehensive Utilization of Saline-Alkali Land, Xinjiang Production and Construction Corps, College of Water Hydraulic and Architectural Engineering, Tarim University, Alar, China; 2 Modern Agricultural Engineering Key Laboratory at Universities of Education Department of Xinjiang Uygur Autonomous Region, Tarim University, Alar, China; 3 Key Laboratory of Tarim Oasis Agriculture, Ministry of Education, Tarim University, Alar, China; 4 Western Agricultural Research Center, Chinese Academy of Agricultural Sciences, Changji, China; 5 Key Laboratory of Northwest Oasis Water-Saving Agriculture, Ministry of Agriculture and Rural Affairs, Shihezi, China

**Keywords:** microbial community, peanut, rhamnolipid-modified biochar, soil enzyme activity, soil quality

## Abstract

**Objective:**

This study aims to elucidate the mechanisms by which rhamnolipid-modified biochar enhances saline soil quality and increases peanut yield, while preliminarily exploring its potential ecological risks. The findings are expected to provide theoretical and technical support for the sustainable improvement of saline soils in arid regions and for the green production of peanuts.

**Methods:**

A field experiment was conducted with three treatments: a control (CK, no biochar application), biochar (BC, application of cotton stalk biochar), and modified biochar (RBC, application of rhamnolipid-modified biochar). The amelioration effects and driving mechanisms of modified biochar on saline soil were investigated by analyzing soil physicochemical properties, microbial community structure, enzyme activities, and peanut yield under different treatments.

**Results and discussion:**

The results showed that, compared with the control, the application of rhamnolipid-modified biochar significantly increased total carbon (by 30.33%-45.20%), total nitrogen (by 28.66%-55.76%), organic carbon (by 244.07%-370.10%), and moisture content (by 25.61%-42.42%) in the 0-20 cm soil layer, while significantly decreasing the total salt content (by 39.40%-56.78%). Furthermore, the RBC treatment activated the activities of soil urease, sucrase, and cellulase. Regarding the microbial community, modified biochar mitigated the inhibitory effect of unmodified biochar on bacterial diversity. It enriched nutrient-rich microbial groups such as Proteobacteria, Acidobacteria, and Firmicutes, as well as salt-tolerant functional bacteria like Neobacillus and Luteitalea, and the fungal phylum Ascomycota. Concurrently, it suppressed the abundance of pathogenic fungi such as Basidiomycota and Colletotrichum, thereby reducing the risk of soil-borne diseases. Random forest analysis identified total carbon, total organic carbon, and soil carbon loading as key environmental factors driving changes in the soil microbial community. Partial least squares path modeling further confirmed that modified biochar improved soil physicochemical properties, activated enzyme activities, subsequently regulated the fungal community, alleviated the suppression of the bacterial community, and fostered the formation of a synergistic microbial network structure. These combined effects ultimately enhanced soil quality and led to a significant 27.57% increase in peanut yield.

## 1 Introduction

Soil salinization, a major environmental constraint on global agricultural production, threatens both land use efficiency and crop growth (Li et al., 2025; Wang et al., 2025). Studies indicate that approximately 20% of irrigated agriculture worldwide is affected by salinization (Zhuang et al., 2021), especially in arid and semi-arid regions, where about 30% of the area faces soil salinization issues (Singh, 2021). Moreover, soil salinization is expanding at an annual rate of 10%, a trend that will steadily erode crop yields and pose a substantial threat to global food security (Chen et al., 2024). Increased soil salinity can damage the soil aggregate structure (Tang et al., 2020), reducing soil nutrient availability (Syed et al., 2021) and accelerate land degradation. Simultaneously, osmotic stress and ionic toxicity suppress root growth, further reducing crop yield (El-Ramady et al., 2024; MaChado and Serralheiro, 2017). Therefore, mitigating soil nutrient depletion and yield reduction caused by soil salinization has become an urgent issue that requires immediate attention.

Biochar is a carbon-rich solid product formed through the high-temperature pyrolysis of organic biomass under anoxic or limited-oxygen conditions (Xia et al., 2024). It possesses a porous structure that enhances soil water retention (Khan et al., 2024), adsorbs harmful ions, and promotes microbial colonization (Palansooriya et al., 2019; Yu et al., 2019). However, the efficiency of biochar in removing exchangeable cations is limited, and its capacity to regulate soil micro-ecosystems under salt stress conditions is insufficient (Han et al., 2021; Zhao et al., 2022). Rhamnolipid is a glycolipid biosurfactant synthesized by Pseudomonas aeruginosa (Kabeil et al., 2025). Its amphipathic structure effectively chelates organic compounds and metal ions, reducing soil salinity and improving the rhizosphere microenvironment to promote plant growth (Cieśla et al., 2018; Liu et al., 2023; Yang et al., 2020). Research has shown that drip application of rhamnolipid achieves desalination rates of 9.7%, 4.5%, and 2.5% for lightly, moderately, and highly saline soils, respectively. Simultaneously, it reshapes the microbial community by significantly enriching plant growth-promoting fungi while inhibiting plant pathogens, thereby enhancing photosynthetic efficiency and crop yield (Liang et al., 2025; Wang et al., 2023). However, the strong emulsifying properties of rhamnolipid may disperse soil aggregates, particularly in sandy soils. Long-term application could reduce soil porosity and water retention capacity, exacerbating soil erosion (Parus et al., 2023). In response, the synergistic effect of biochar and rhamnolipid enhances the dispersibility and adsorption capacity of biochar while improving its alkalinity resistance (Chebbi et al., 2022; Hu et al., 2023). This synergistic mechanism provides new insights for the remediation of saline soils.

In China, peanuts are a crucial oil-producing crop, with both a large planting area and high yield that rank among the highest globally (Lu et al., 2024). According to statistics, the national peanut planting area reached 4.83 × 10^7^ hm^2^ in 2024, with total production increasing to 1.90 × 10^7^ tons. However, peanuts are highly sensitive to soil salinity, and salt stress inhibits the activity of symbiotic nitrogen-fixing microorganisms, such as Rhizobium and Burkholderia, which reduces the abundance of microbial communities involved in the carbon, nitrogen, and phosphorus cycles in the soil, thereby exacerbating nutrient absorption barriers in peanuts (Ben Gaied et al., 2024; Sharma et al., 2016). Due to the ongoing effects of soil salinization, the average peanut yield in Xinjiang is 28.5% lower than the national average, severely limiting the sustainable development of the regional peanut industry and becoming a major bottleneck to agricultural efficiency and farmers’ income growth (Cui et al., 2018).

Modified biochar, due to its unique physicochemical properties and ecological functions, has shown great potential in regulating crop salt tolerance and reshaping soil microbial communities. However, existing studies have not systematically revealed the regulatory mechanisms of biochar modification on the physicochemical properties and microbial community structure in saline soils, nor clarified the pathways linking soil quality improvement with peanut yield formation. Therefore, this study systematically explores the “soil-microbe-crop” cascade regulatory mechanism, discovering that modified biochar affects soil physicochemical properties, microbial communities, and peanut yield. Additionally, partial least squares analysis was used to examine the relationship between peanut yield and the soil microenvironment, thereby evaluating the amelioration effects of modified biochar on saline soils. The goal is to explore a strategy that can both alleviate soil salt stress and improve soil quality, while also improving peanut growth, yield, and quality through the regulation of soil micro-ecosystems. This will have significant practical implications for enhancing the agricultural productivity of saline soils in southern Xinjiang.

## 2 Materials and methods

### 2.1 Study area

**Figure 1:**
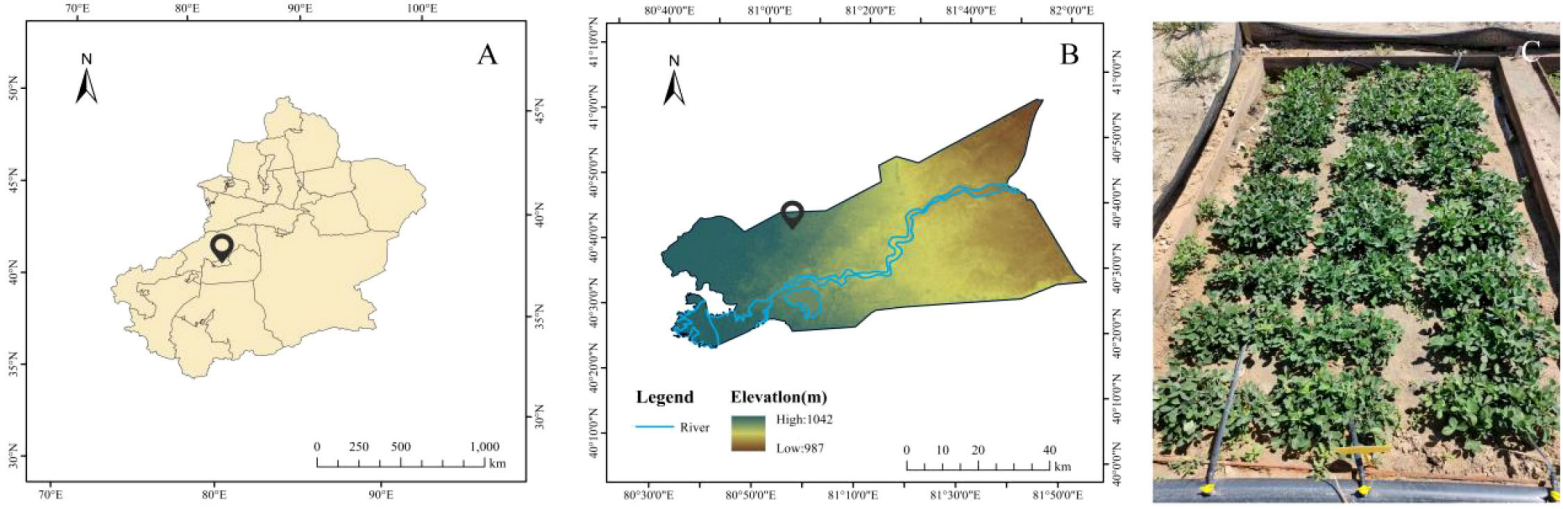
Study sites. **(A)** is a regional map of Xinjiang, China; **(B)** is a regional map of Aral City, Xinjiang; **(C)** depicts the specific experimental planting environment.

The experiment was conducted at the field experimental base of the Modern Agricultural Academician and Expert Workstation in Aral, Xinjiang (81.17°E, 40.32°N, elevation 1100 m) (**Figure 1**). The experimental area has a warm temperate extreme continental arid desert climate, characterized by significant dryness and high evaporation rates. The average annual precipitation is less than 100 mm, with evaporation exceeding 2000 mm, and the diurnal temperature variation can reach up to 20 °C. The soil texture is sandy loam, having a bulk density of 1.60 g·cm^-3^, a field water holding capacity of 21%, and soil electrical conductivity and pH values ranging from 3.05 to 3.97 ms·cm^-1^ and 7.70 to 7.84, respectively. The total carbon, total nitrogen, and organic carbon content in the soil are 17.98 g·kg^-1^, 0.61g·kg^-1^, and 1.53 g·kg^-1^, respectively, showing overall characteristics of infertility and prominent salinization.

### 2.2 Biochar preparation

Cotton stalks were subjected to a series of treatments to prepare biochar (BC) and rhamnolipid-modified biochar (RBC). First, the cotton stalks were dried at 105 °C, then heated in steam at 170 °C for 1 hour, and subsequently pretreated with 7.0% (v/v) sulfuric acid to treat the lignocellulosic material. The pretreated residue was then pyrolyzed at 500 °C for 2 hours, ground into a fine powder, and passed through a 2 mm sieve to produce cotton stalk biochar. The preparation of rhamnolipid-modified biochar involved mixing cotton stalk biochar with rhamnolipid at a solution ratio (rhamnolipid:water = 2:8) for impregnation, followed by equilibrium at room temperature for 24 hours to obtain the modified biochar. The physicochemical properties of the tested biochars are shown in **Table 1**. Rhamnolipid was provided by Yuyuan Biotechnology Co., Ltd. in Xinzhen City, with a purity of 98%.

**Table 1:** Basic physical and chemical properties of biochar.

Biochar	Specific surface area (m^2^·g^-1^)	Average pore size (nm)	C (%)	O (%)	N (%)
BC	68.29	5.88	44.44	25.38	1.33
RBC	55.85	7.10	34.81	34.28	1.68

### 2.3 Experimental design

The experiment included three treatments: control (CK), biochar (BC), and modified biochar (RBC), with three replicates per treatment, totaling 9 test pits. Each test pit had an area of 6.6 m^2^ (2.0 m × 3.3 m) and a vertical depth of 3 m. The test pits were separated by cement walls and steel plates, with the surfaces coated with asphalt to prevent leakage. Biochar was uniformly applied to the soil surface and mixed into the 0–20 cm soil layer with a shovel. A dose of 4 kg·m^-2^ of biochar was applied to each test pit, based on two considerations: first, the tested soil has very low organic carbon and relatively high salinity, requiring a higher dose for rapid improvement; second, cotton stalk resources are abundant and underutilized in southern Xinjiang, making the high-dose single application both resource-based and economically feasible. This aims to reconstruct the degraded soil matrix and promote long-term soil quality improvement. Peanut planting was initiated after the biochar stabilized for one week.

**Figure 2:**
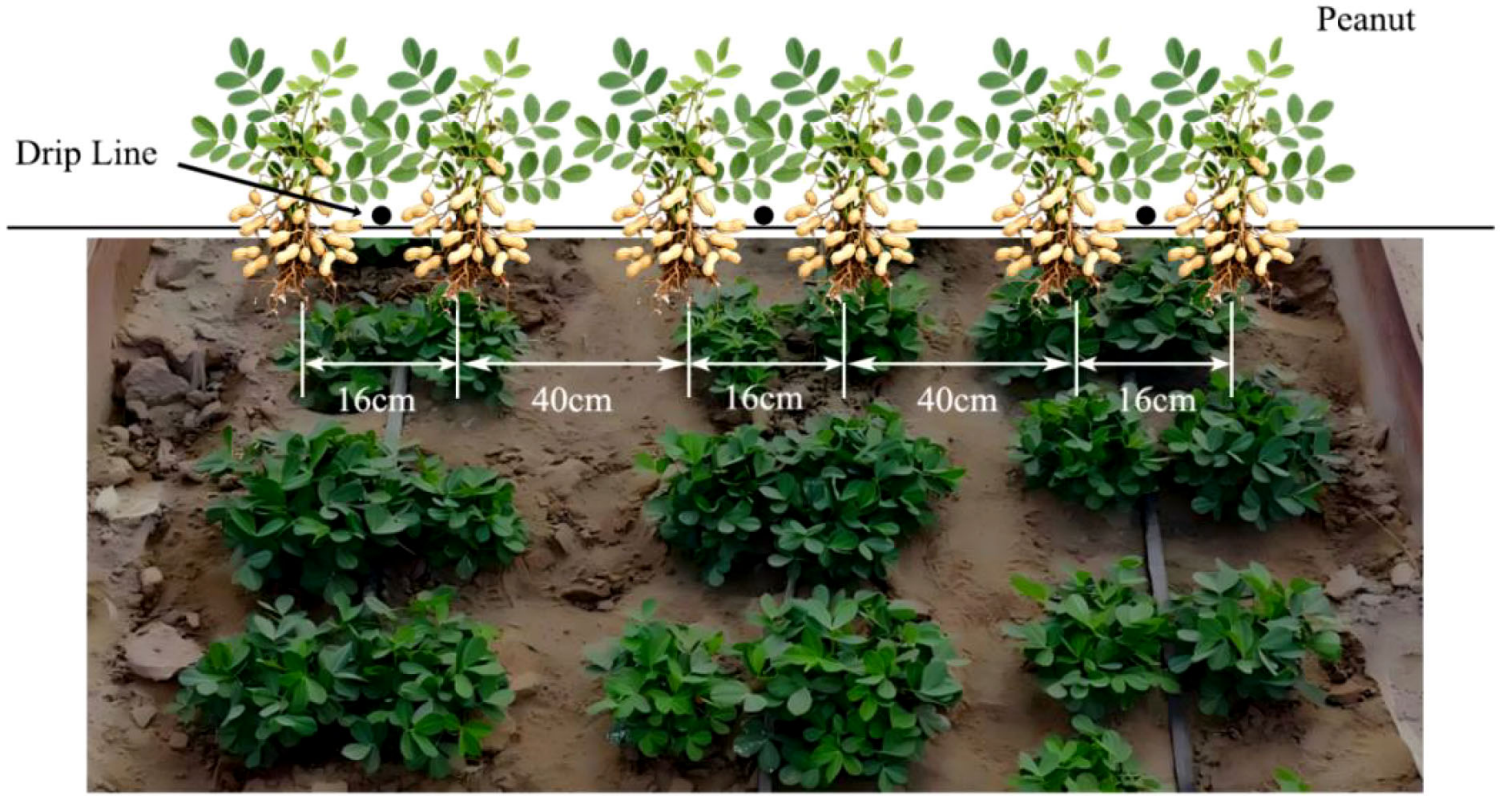
Schematic diagram of peanut cultivation pattern.

The peanut variety used was “Xin Hua15”, A three-tube six-row under-mulch drip irrigation system was employed, with wide rows of 40 cm, narrow rows of 16 cm, and plant spacing of 15 cm (as shown in **Figure 2**). Built in patch type drip tapes were used, with emitters spaced at 30 cm intervals, rated flow of 3.0 L·h^-1^, and operating pressure of 0.1 MPa. Fertilizers were applied through fertigation during the peanut growing season. Starting from the flowering and pod-setting stage, urea (N = 45%) at 300 kg·hm^-2^, monoammonium phosphate (N:P_2_O_5_ = 12:61) at 510 kg·hm^-2^, and potassium sulfate (K_2_O = 35.8%) at 225 kg·hm^-2^ were applied. Additionally, 75 kg·hm^-2^ of chelated calcium (Ca=12%) was applied during the pod-filling and full fruit maturity stages. Field management practices were strictly followed according to the local agricultural production model, ensuring consistency across all treatments.

### 2.4 Test indicators and methods

#### 2.4.1 Soil sample collection

Mixed soil samples from the 0–20 cm soil layer were collected at each measurement pit. Soil enzyme activities and microbial community structure were sampled at the peanut pod maturity stage, while soil moisture content, total salt, total carbon, total nitrogen, and organic carbon were measured throughout the entire growing season.

#### 2.4.2 Determination of soil physical and chemical properties

The moisture content was measured by the drying method. Fresh soil samples were placed in an oven at 105 °C to dry, then cooled to room temperature and weighed immediately after achieving constant weight. The electrical conductivity of the soil extract (water:soil = 5:1) was measured using a DDP-210 portable conductivity meter. The relationship between soil salinity and electrical conductivity was calibrated using the drying method, and total dissolved solids was calculated using the Equation 1. Soil organic carbon was measured using a total organic carbon analyzer (Elementarenviro TOC). Total carbon and total nitrogen were measured with an elemental analyzer (Elementarvario isotope select). Soil enzyme activities, including urease (RUE), alkaline phosphatase (AKP), catalase (CAT), sucrase (SUC), and cellulase (SCL), were measured using kits provided by Shanghai Lien Bio Co., Ltd. The kits were supplied by Nanjing Mofan Biotechnology Co., Ltd., and enzyme activity was measured with a full-wavelength microplate reader (SpectraMax 190, Molecular Devices, USA).

(1) $$\text{y}=0.0032{EC}_{5:1}-1.7739$$

In the equation: y represents the total dissolved solids in soil (g·kg^-1^), and EC_5:1_ is the electrical conductivity of the soil extract (μs/cm) at 25 °C.

#### 2.4.3 Soil microbial community structure

The total DNA of the microbial community in the soil samples was extracted using the E.Z.N.A.^®^ Soil DNA Kit (Omega Bio-tek, Norcross, GA, U.S.). For analyzing the bacterial community structure, full-length universal primers 27F (AGRGTTYGATYMTGGCTCAG) and 1492R (RGYTACCTTGTTACGACTT) targeting the 16S rRNA gene were employed for amplification. Conversely, amplification of the fungal community structure was performed using full-length universal primers ITS1F (5’-CTTGGTCATTTAGAGGAAGTAA-3’) and ITS2R (5’-GCTGCGTTCTTCATCGATGC-3’), which are specific to the ITS rDNA region. The amplified products were purified after 2% agarose gel electrophoresis using the AxyPrep DNA Gel Extraction Kit (Axygen Biosciences, Union City, CA, U.S.) according to the manufacturer’s instructions. Following the protocols provided by Pacific Biosciences, the SMRTbell library was constructed from the amplified DNA through blunt-end ligation. After assigning unique barcode sequences to each sample, mixtures of equal quality were pooled together. The resulting amplicon mixture was subsequently utilized to build the sequencing library with the Pacific Biosciences SMRTbellTM Template Prep Kit 1.0, followed by sequencing on the PacBio Sequel II platform.

#### 2.4.4 Peanut production

Harvesting begins when the peanut leaves turn yellow, with half of the leaves falling off, and the pod shells harden and display distinct markings. All peanuts from each experimental pit were fully harvested, air-dried, and then weighed.

#### 2.4.5 Soil quality index calculation

A linear calculation model was used to convert soil salinity and quality indicators into scores between 0 and 1. In this study, the “the more the better” type indicator scoring Equation 2 and the “the less the better” type indicator scoring Equation 3 were applied.

(2) $$S_{\text{i}}=\frac{{\text{x}}_{\text{i}}-{\text{x}}_{\text{min}}}{{\text{x}}_{\text{max}}-{\text{x}}_{\text{min}}}$$

(3) $$S_{\text{i}}=\frac{{\text{x}}_{\text{max}}-{\text{x}}_{\text{i}}}{{\text{x}}_{\text{max}}-{\text{x}}_{\text{min}}}$$

In the equation: S_i_ represents the linear indicator score (0-1), X_i_ represents the measured value of the indicator, X_max_ represents the maximum value of the indicator, and X_min_ represents the minimum value of the indicator.

To calculate the soil quality index (SQI) for the 0–20 cm soil layer, principal component analysis was performed on the 12 soil indicators measured in this study. The common factor variance for each indicator was extracted, and the ratio of the individual communality to the sum of communalities of all indicators was calculated to determine the weight. This objective weighting method, based on the internal variance of the dataset, reduces the bias inherent in expert-assigned weights and ensures that indicators with higher sensitivity to the treatments are appropriately emphasized. The SQI was calculated using Equation 4, where a higher SQI value indicates better soil quality.

(4) $$SQI=\sum_{\text{i}=1}^{\text{n}}W_{\text{i}}S_{\text{i}}$$

In the equation: SQI is the soil quality index, Wi is the weight of the i-th indicator, Si is the score of the i-th indicator, and n is the number of indicators in each dataset, where n = 12 in this study.

### 2.5 Data processing

The experimental data were organized using Microsoft Excel 2016. One-way analysis of variance (ANOVA), principal component analysis, and correlation analysis (P < 0.05) were conducted using IBM SPSS 26.0. Microbial community structure analysis was performed using the Biozeron Delivery System, R (version 4.4.3), and the RStudio Integrated Development Environment (IDE). Partial Least Squares Path Modeling (PLS-PM) analysis was carried out using the R package plspm (version 0.5.0). Finally, results were visualized using the “ggplot2” package, Origin 2024, and Microsoft PowerPoint 2016.

## 3 Results and analysis

### 3.1 Soil microbial community diversity analysis

**Figure 3:**
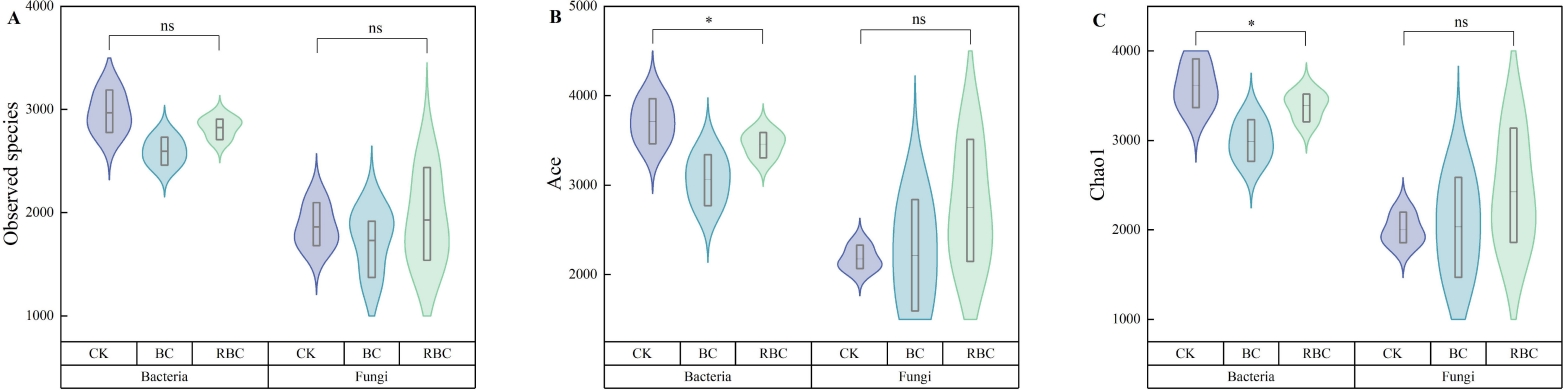
Soil microbial alpha abundance and diversity. Changes in the Observed species index **(A)**, ACE index **(B)**, and Chao1 index **(C)** of bacteria and fungi in soil under different biochar treatments. * indicates significant differences between treatments (P<0.05), while ns indicates no significant differences between treatments (P>0.05).

The alpha diversity and abundance of soil microorganisms are shown in **Figure 3**. A total of 54,539 and 18,453 valid sequences of the bacterial 16S rRNA gene and fungal ITS gene were obtained, with coverage greater than 97%. The sequencing depth effectively covered most of the microorganisms in the samples, reflecting the true status of the soil microbial community. In the bacterial community, relative to the CK treatment, the BC treatment decreased the observed species index by 4.80% – 12.61%, ACE index by 6.84% – 17.56%, and Chao1 index by 6.38% – 17.28%, with significant reductions in the ACE and Chao1 indices. In contrast, the reductions in the observed species index, ACE index, and Chao1 index for the RBC treatment were generally lower than those for the BC treatment. In the fungal community, the addition of rhamnolipid-modified biochar promoted a 3.53% – 7.05% increase in the observed species index, a 1.83% – 26.28% increase in the ACE index, and a 1.59% – 21.14% increase in the Chao1 index. In contrast, the BC treatment showed smaller increases than the RBC treatment.

**Figure 4:**
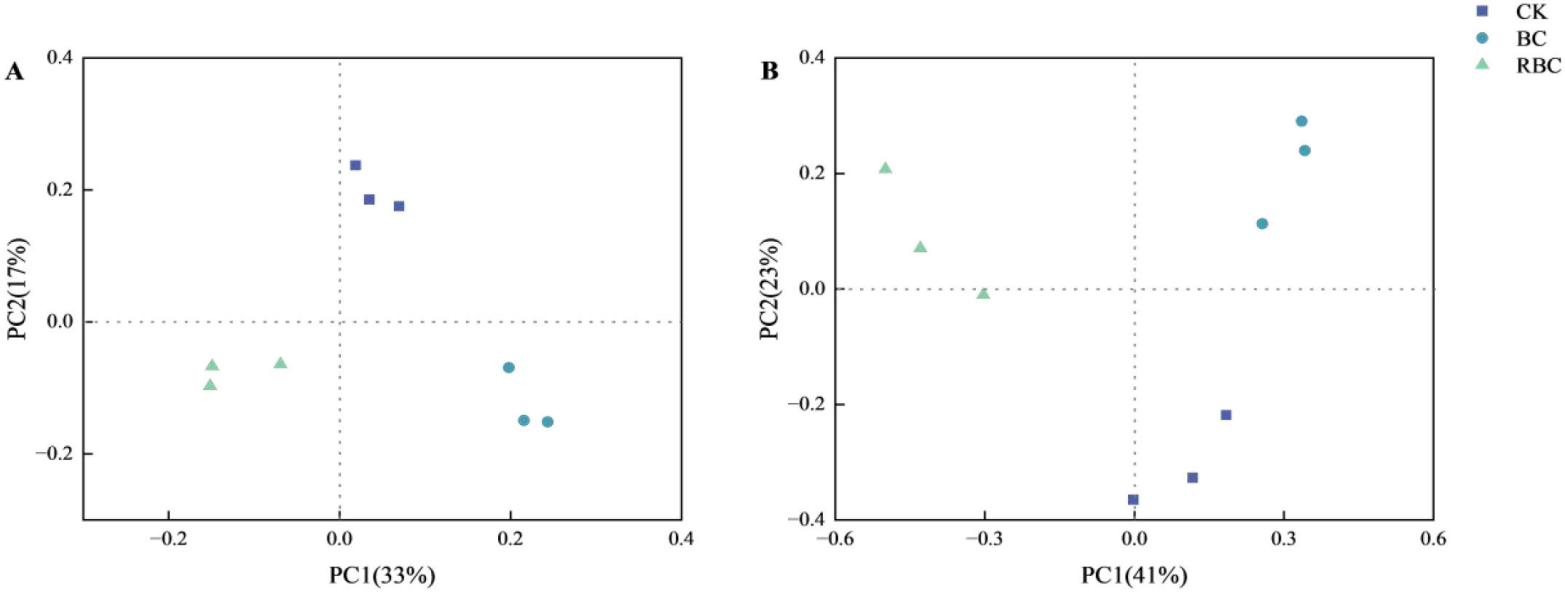
Soil microbial beta diversity. Beta diversity of bacteria **(A)** and fungi **(B)** in soil under different biochar treatments.

The beta diversity of soil microorganisms is illustrated in **Figure 4**. For bacteria, the cumulative percentage variance in species explained by the first and second principal coordinates is 33% and 17%, respectively, while for fungi, these values are 41% and 23%, respectively. In the RBC treatment, both bacterial and fungal communities are distributed in the negative direction of PC1, while the BC treatment is located in the positive direction of PC1. Therefore, there is a clear separation in microbial community structure between the CK, BC, and RBC treatments, indicating that the addition of rhamnolipid-modified biochar significantly induces changes in both bacterial and fungal communities.

### 3.2 Soil bacterial and fungal community composition analysis

**Figure 5:**
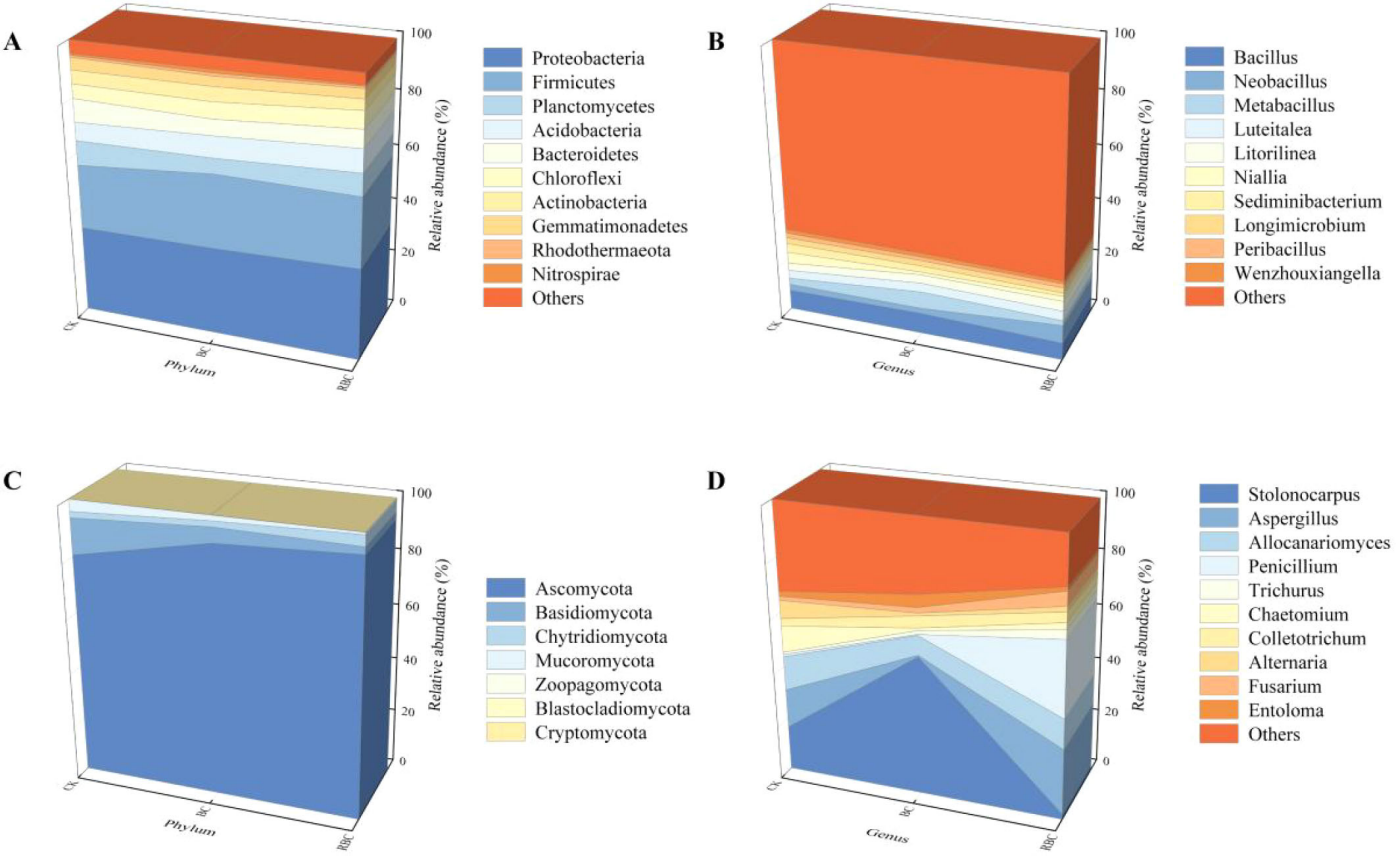
Relative abundance of soil microbial communities. Relative abundance of dominant bacterial and fungal species at the phylum level **(A, C)** and genus level **(B, D)** in soil under different biochar treatments.

The soil bacterial community structure consists of 34 phyla, and the top 10 species based on relative abundance at the phylum (**Figure 5A**) and genus (**Figure 5B**) levels are shown, with other groups classified as “Others”. At the phylum level, Proteobacteria (25.78% – 38.56%) exhibited the highest relative abundance, followed by Firmicutes (15.23% – 37.48%), Planctomycetes (4.45% – 10.09%), Acidobacteria (5.82% – 8.63%), Bacteroidetes (4.37% – 11.60%), Chloroflexi (4.42% – 7.29%), Actinobacteria (3.25% – 6.43%), Gemmatimonadetes (2.69% – 4.80%), Rhodothermaeota (0.57% – 1.41%), and Nitrospirae (0.35% – 1.96%). Compared to CK, the rhamnolipid-modified biochar (RBC) treatment increased Proteobacteria by 1.63%, Firmicutes by 3.35%, Acidobacteria by 17.39%, and Chloroflexi by 12.8%, with the increase in Firmicutes being smaller than that in the BC treatment. At the genus level, Bacillus (3.61% – 9.71%) had the highest relative abundance, followed by Neobacillus (1.63% – 8.96%), Metabacillus (1.12% – 8.91%), Luteitalea (2.63% – 3.95%), Litorilinea (2.45% – 4.21%), Niallia (0.71% – 10.01%), Sediminibacterium (0.37% – 5.90%), Longimicrobium (1.48% – 2.50%), Peribacillus (0.63% – 2.54%), and Wenzhouxiangella (0.88% – 2.20%). Compared to CK, the RBC treatment increased Luteitalea by 13.35% and Neobacillus by 176.09%, with both increases being greater than those observed in the BC treatment.

The soil fungal community structure consists of 8 phyla, and the top 10 species based on relative abundance at the phylum (**Figure 5C**) and genus (**Figure 5D**) levels are shown, with other groups classified as “Others.” At the phylum level, Ascomycota (67.66% – 97.86%), Basidiomycota (0.79% – 25.44%), Chytridiomycota "Others" (0.07% – 9.14%), Mucoromycota (0.55% – 5.06%), Zoopagomycota (0.0037% – 0.063%), Blastocladiomycota (0.00% – 0.074%), and Cryptomycota (0.00% – 0.0074%) were observed. Compared to CK, the rhamnolipid-modified biochar (RBC) treatment increased Ascomycota by 15.01%, Chytridiomycota by 76.30%, and Zoopagomycota by 755.29%, with all increases being greater than those observed in the BC treatment. At the genus level, Stolonocarpus (0.66% – 42.68%) had the highest relative abundance, followed by Aspergillus (0.36% – 31.43%), Allocanariomyces (3.03% – 19.02%), Penicillium (0.12% – 47.28%), Trichurus (0.93% – 11.85%), Chaetomium (0.44% – 15.26%), Colletotrichum (0.83% – 6.44%), Alternaria (0.27% – 14.35%), Fusarium (1.21% – 8.58%), and Entoloma (0.56% – 7.29%). Compared to CK, the RBC treatment increased Aspergillus by 83.46%, Fusarium by 264.77%, and Penicillium by 3453.79%.

### 3.3 Microbial community co-occurrence network analysis

**Figure 6:**
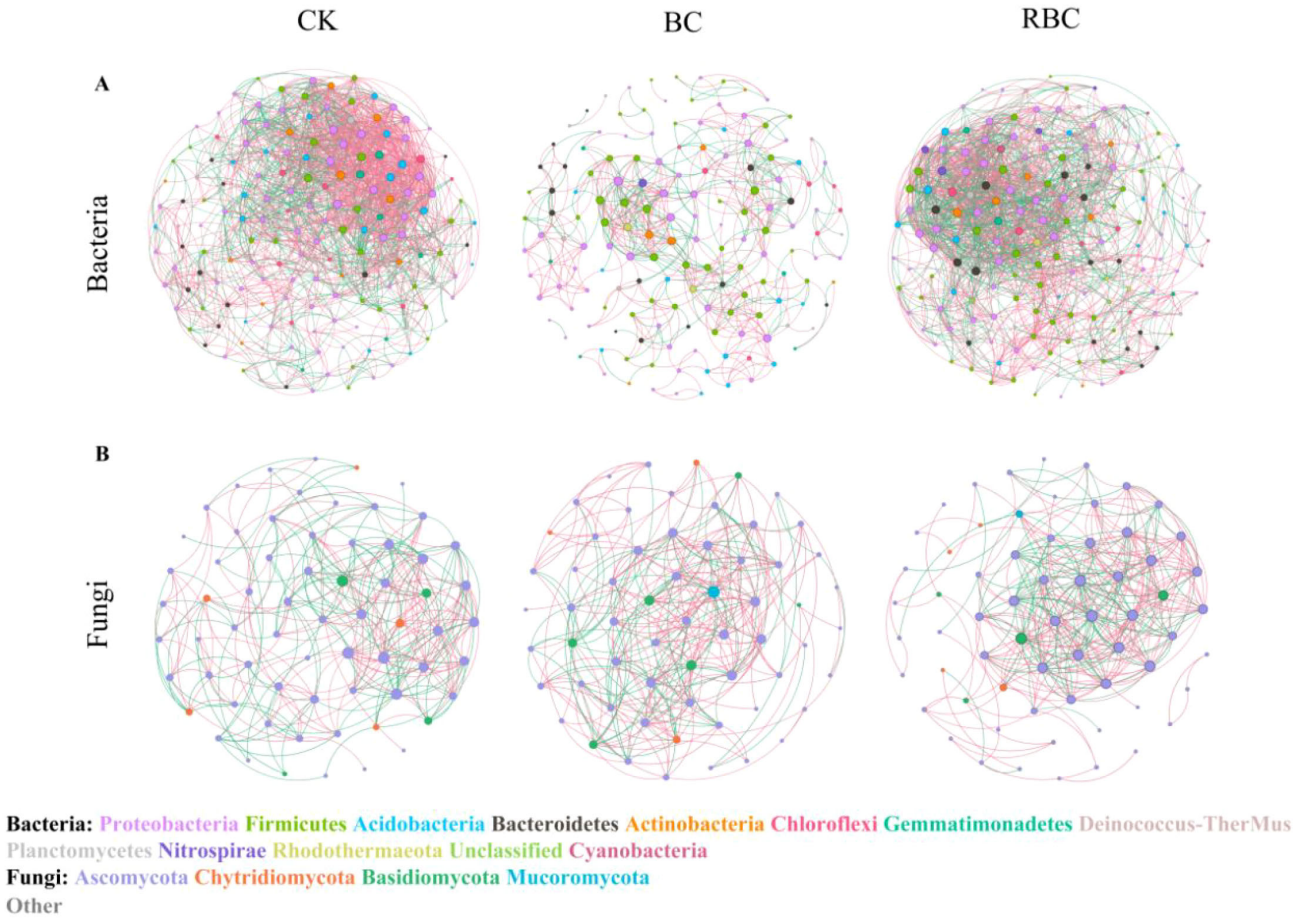
Bacterial and fungal co-occurrence network diagram. Co-occurrence network interactions of soil bacterial **(A)** and fungal **(B)** communities under different biochar treatments. The nodes in the networks represent taxa at the phylum level. The edges indicate statistically significant correlations between nodes (R > 0.7, P < 0.05), with red and green edges representing positive and negative correlations, respectively. The size of each node is proportional to its number of connections (node degree).

To investigate the reactions of microbial co-occurrence associations to different biochar treatments, a co-occurrence network analysis was performed (**Figure 6**), with topological parameters shown in **Table 2**. The modularity index of all co-occurrence networks exceeded 0.4, demonstrating that the networks exhibit notable modular structural characteristics. In the bacterial co-occurrence network, compared to CK, the BC treatment reduced the total number of links, average degree, and average clustering coefficient by 67.21%, 64.67%, and 1.44%, respectively. In contrast, for the RBC treatment, the total number of links, average degree, and average clustering coefficient were reduced by only 1.11%, 2.85%, and 5.95%, respectively, while the total number of nodes and average path length increased by 1.76% and 1.81%, respectively, compared to CK. Moreover, the core bacterial phyla in the co-occurrence network were Proteobacteria, Firmicutes, Acidobacteria, Bacteroidetes, Actinobacteria, Chloroflexi, and Gemmatimonadetes, accounting for about 90% of the total nodes in the network. These taxa were key drivers of bacterial community interactions. In the fungal co-occurrence network, the effect of biochar addition showed the opposite trend compared to bacteria. The BC treatment increased the total number of nodes, total number of links, and average degree by 1.67%, 0.34%, and 6.92%, respectively. In contrast, the promoting effect of the RBC treatment was more significant, with the total number of nodes, total number of links, and average degree increasing by 6.67%, 12.93%, and 14.69%, respectively, compared to CK. In the fungal co-occurrence network, the core fungal phyla were Ascomycota, Chytridiomycota, and Basidiomycota, accounting for about 90% of the total nodes in the network. These taxa were the dominant drivers of fungal community interactions.

**Table 2:** Based on topological parameter.

Treatment		Total number of nodes	Total number of links	Average degree	Average clustering coefficient	Average path length	Modularity
Bacteria	CK	167	1711	20.491	0.554	2.759	0.483
	BC	155	561	7.239	0.546	4.846	0.692
	RBC	170	1692	19.906	0.521	2.809	0.404
Fungi	CK	60	294	9.046	0.568	2.955	0.473
	BC	61	295	9.672	0.611	3.308	0.405
	RBC	64	332	10.375	0.562	3.087	0.484

### 3.4 Changes in soil physicochemical properties

**Figure 7:**
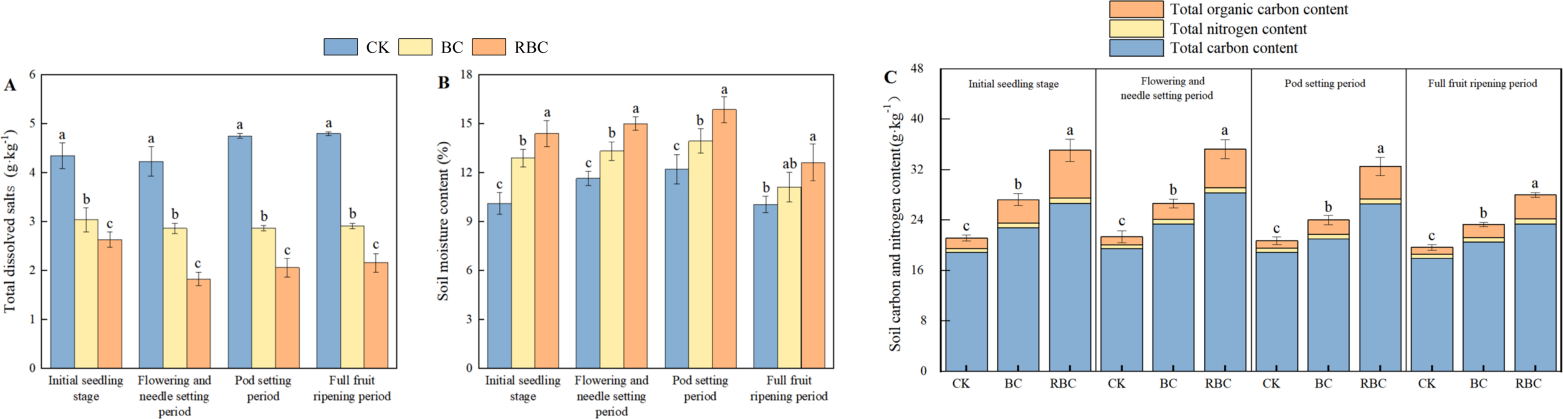
Changes in soil physicochemical properties. Changes in total dissolved solids **(A)**, moisture content **(B)**, and soil carbon and nitrogen content **(C)** at different growth stages under different biochar treatments. Different letters indicate significant differences between treatments within the same growth stage (P < 0.05).

The variations in soil physicochemical properties over time and space are presented in **Figure 7**. Each indicator exhibits distinct dynamic trends during the growing season. Soil carbon, nitrogen, and moisture content first increase and then decrease during the growth process, while total dissolved solids shows distinct phase fluctuations. Compared to CK, the RBC treatment markedly elevated soil carbon and nitrogen content, with total carbon, total nitrogen, and organic carbon increasing by 30.33% – 45.20%, 28.66% – 55.76%, and 244.07% – 370.10%, respectively. It also promoted an increase in soil moisture content (25.61% – 42.42%) and a reduction in total dissolved solids (39.40% – 56.78%). In contrast, the BC treatment showed a smaller improvement in soil physicochemical properties compared to the RBC treatment, with increases in total carbon, total nitrogen, and organic carbon of 11.21% – 20.68%, 10.98% – 24.48%, and 88.14% – 115.55%, respectively.

### 3.5 Changes in soil enzyme activity

**Figure 8:**
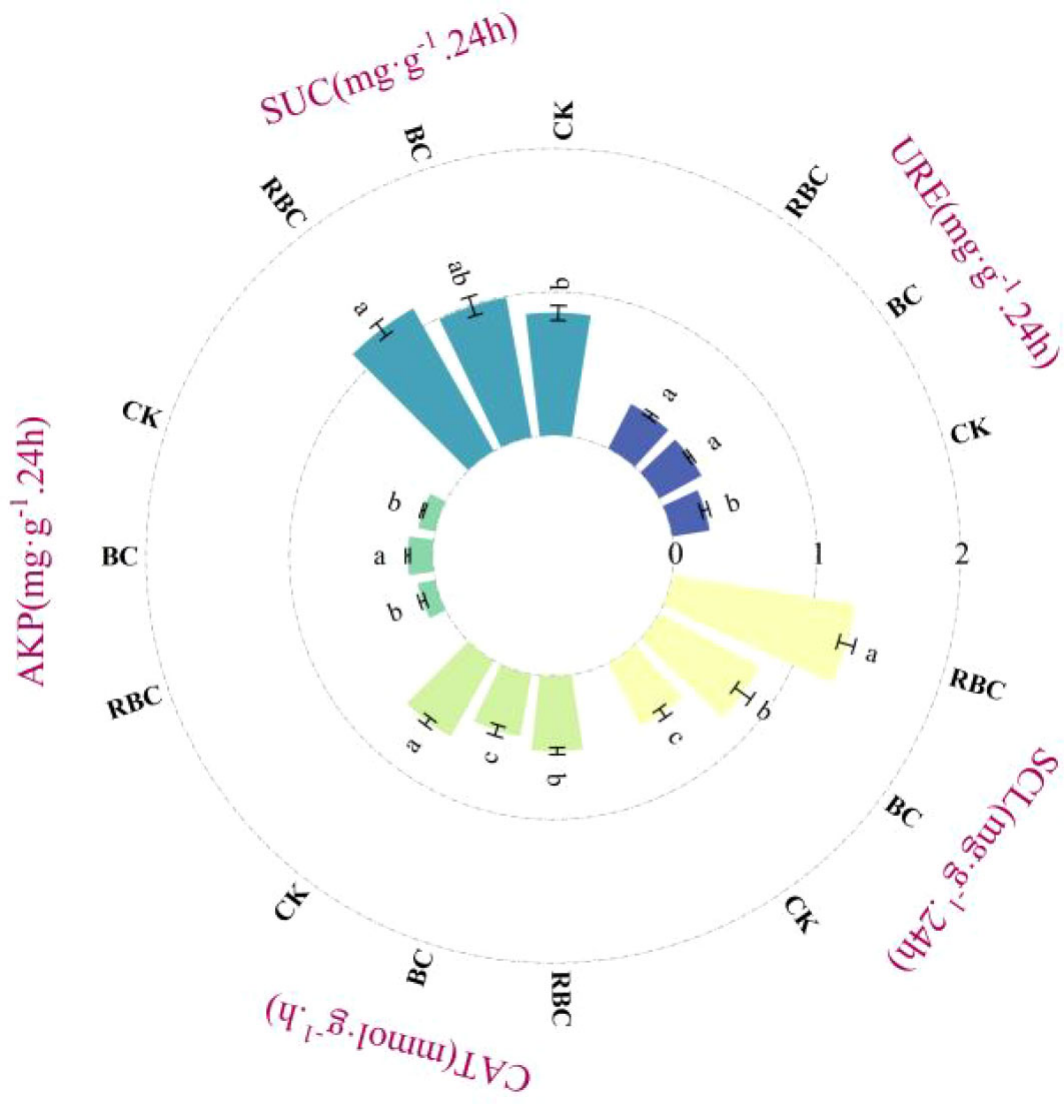
Changes in soil enzyme activity. Changes in soil URE (urease), SUC (sucrase), AKP (alkaline phosphatase), CAT (catalase), and SCL (cellulase) activity under different biochar treatments.

The different responses of soil enzyme activity are shown in **Figure 8**. Compared to CK, the RBC treatment increased RUE by 31.67%, SUC by 33.62%, AKP by 3.61%, and SCL by 160.66%, while decreasing CAT activity by 14.13%. In contrast, the biochar treatment (BC) had a weaker effect on soil enzyme activity regulation compared to the RBC treatment. The increases in RUE, SUC, and SCL activity were lower in the BC treatment than in the RBC treatment, but the increase in AKP activity (27.72%) was markedly greater than that in the RBC treatment. Meanwhile, CAT activity in the BC treatment decreased by 27.72% compared to CK, a greater decrease than in the RBC treatment.

### 3.6 Changes in soil quality index and peanut yield

The soil quality index (SQI) for the 0–20 cm soil layer was calculated using the full dataset (12 indicators) (**Table 3**). The “more is better” scoring model was applied to indicators positively affecting soil properties, including SMC, TC, TN, TOC, RUE, SUC, AKP, CAT, SCL, Bacteria, and Fungi. The “more is better” scoring model was applied to indicators negatively affecting soil properties, including TDS. The SQI for the BC and RBC treatments increased significantly by 108.70% and 278.26%, respectively, compared to CK (P < 0.05). The peanut yield for the BC and RBC treatments increased significantly by 13.22% and 27.57%, respectively, compared to CK (P < 0.05). Furthermore, linear regression analysis demonstrated a significant positive correlation between SQI and peanut yield, with an R^2^ of 0.9118. .

**Table 3:** Peanut yield and soil quality index.

Soil quality evaluation indicators			Relationship between yield and soil quality index		
Indicator	Common factor variance	Weight	Treatment	Yield(kg·hm^-2^)	SQI
SMC	0.938	0.091	CK	5089.53 ± 192.96c	0.23 ± 0.01c
TDS	0.912	0.089	BC	5762.47 ± 135.79b	0.48 ± 0.03b
TC	0.927	0.090	RBC	6492.69 ± 272.75a	0.87 ± 0.06a
TN	0.977	0.095			
TOC	0.907	0.088	Linear fitting		
RUE	0.952	0.093			
SUC	0.967	0.094		Y = 1828.4x + 4880.4	
AKP	0.752	0.073		R^2^ = 0.9118	
CAT	0.601	0.059		P<0.01	
SCL	0.914	0.089			
Bacteria	0.639	0.062			
Fungi	0.779	0.076			

The listed data are presented as mean ± standard deviation (n=3), with different letters indicating significant differences between treatments (P < 0.05).

### 3.7 Soil microbial community structure driver

**Figure 9:**
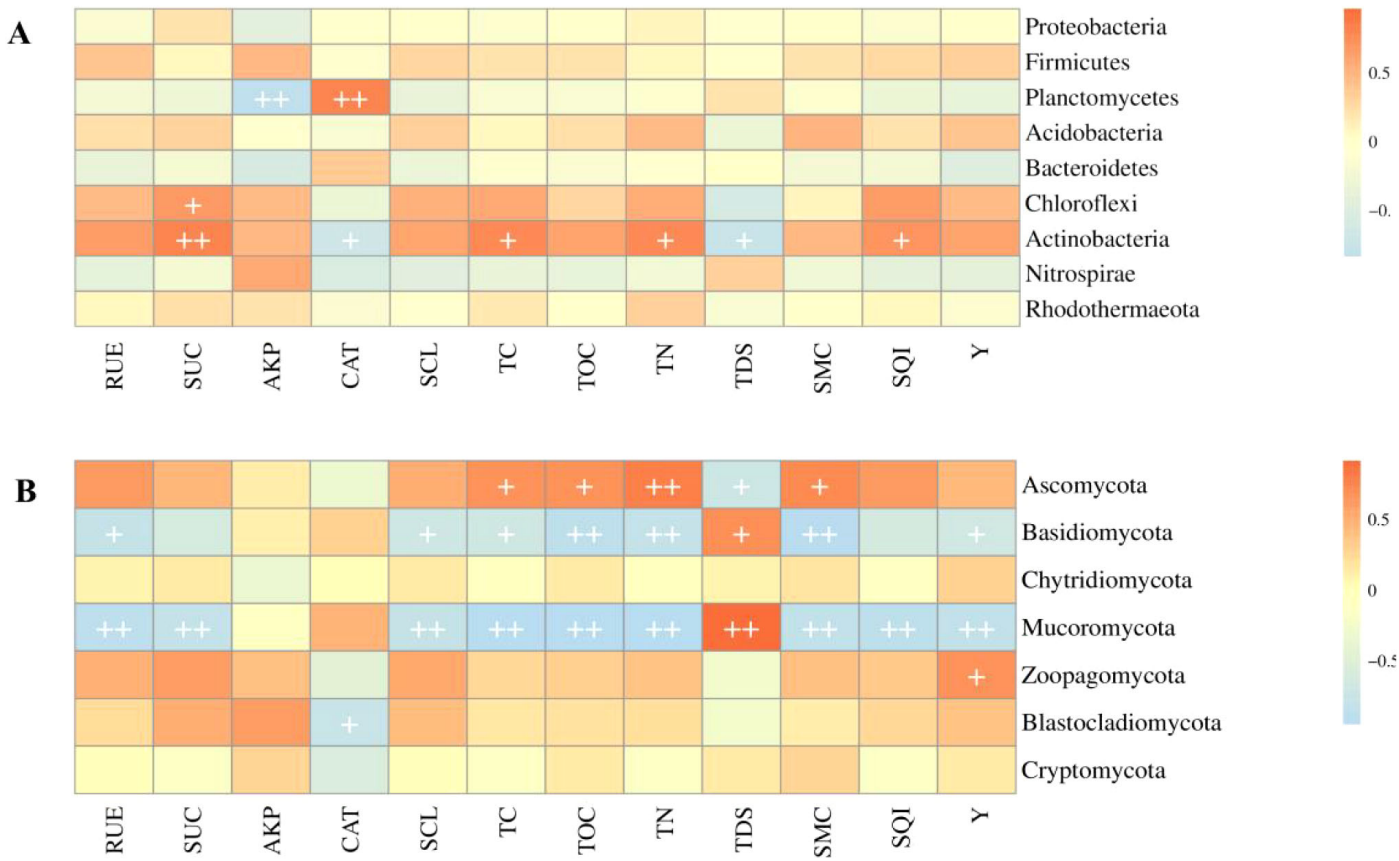
Correlation analysis between soil environmental factors and major microbial species. Correlation analysis of soil bacterial **(A)** and fungal **(B)** species under different biochar treatments. Soil environmental factors include urease (RUE), sucrase (SUC), alkaline phosphatase (AKP), catalase (CAT), cellulase (SCL), total carbon (TC), organic carbon (TOC), total nitrogen (TN), total dissolved solids (TDS), moisture content (SMC), soil quality index (SQI), and yield (Y).

The correlations between dominant bacterial taxa and the soil environment are shown in **Figure 9A**. Specifically, Planctomycetes exhibited a highly significant negative association with AKP and a highly significant positive association with CAT. Chloroflexi exhibited a significant positive correlation with SUC. Actinobacteria exhibited significant positive correlations with SUC, TC, TN, and SQI, and significant negative correlations with TDS and CAT. The correlation analysis between dominant fungal taxa and the soil environment is shown in **Figure 9B**. Ascomycota showed significant positive correlations with TC, TOC, TN, and SMC, and a significant negative correlation with TDS. Basidiomycota showed significant negative correlations with RUE, SCL, TC, TOC, TN, SMC, and Y, and a significant positive correlation with TDS. Mucoromycota showed significant negative correlations with RUE, SUC, SCL, TC, TOC, TN, SMC, SQI, and Y, and a highly significant positive correlation with TDS. Zoopagomycota showed a significant positive correlation with Y, while Blastocladiomycota showed a significant negative correlation with CAT.

**Figure 10:**
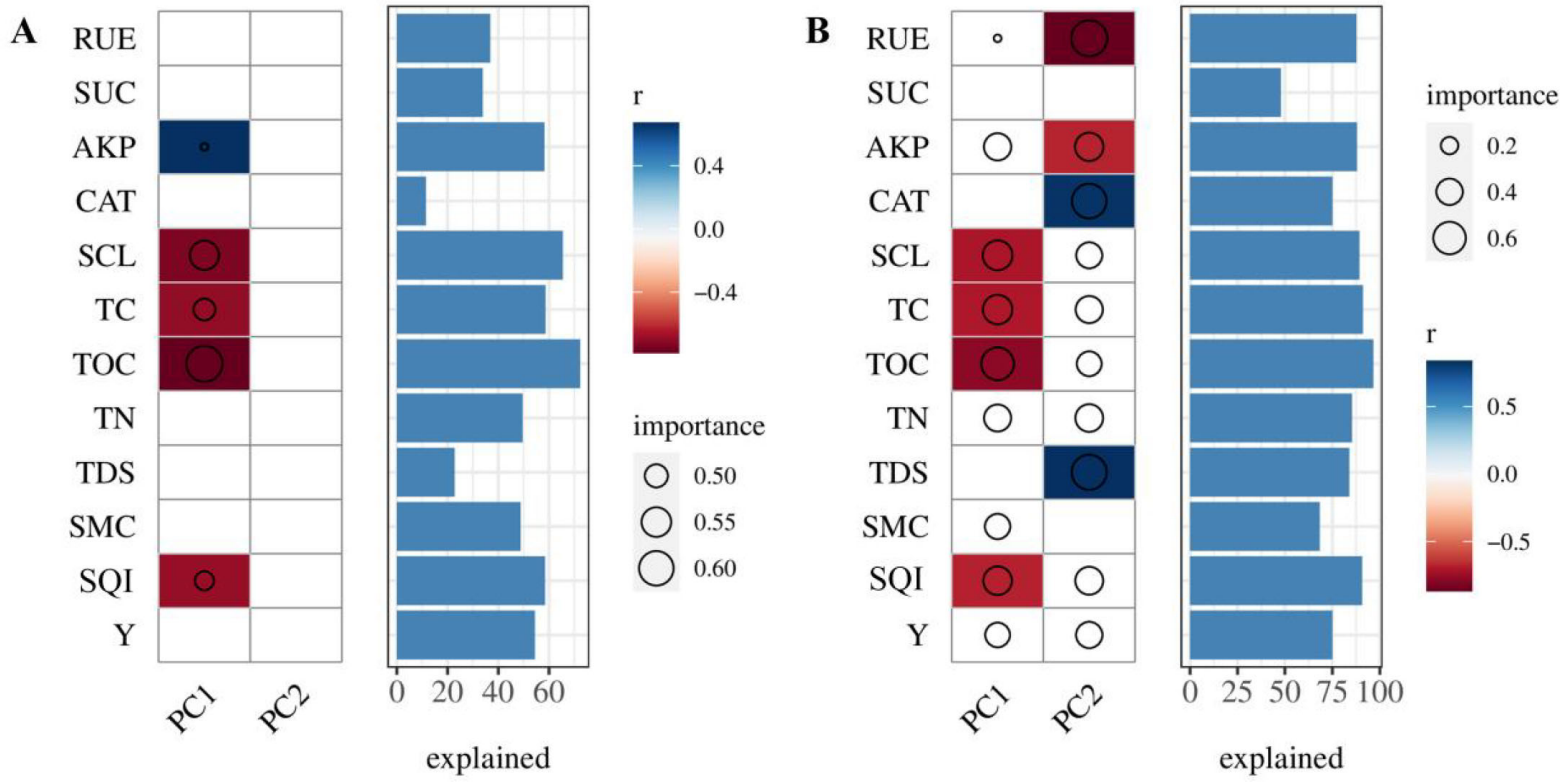
Correlation analysis between soil environmental factors and PC1-2. Correlation analysis of soil bacterial **(A)** and fungal **(B)** species under different biochar treatments. The color blocks represent environmental factors significantly correlated with microbial PC1 and PC2 scores (P < 0.05) based on Spearman correlation (blank if not significant). Circles indicate the size of key factors, and bar charts represent the proportion of community variation explained by each factor. The same applies to specific soil environmental factors.

After performing PCA dimensionality reduction on the microbial data, the relationship between environmental factor data and the microbial community PC1 and PC2 scores was analyzed using Spearman correlation and random forest models (**Figure 10**). In the bacterial community, AKP, SCL, TC, TOC, and SQI are the key factors driving the community structure of PC1, with TOC and SCL explaining 72.35% and 65.58% of the variation in microbial community changes, respectively. In the fungal community, RUE, AKP, SCL, TC, TOC, TN, SMC, SQI, and Y are the primary factors associated with PC1 variation, while RUE, AKP, CAT, SCL, TC, TOC, TN, TDS, SQI, and Y are the primary factors related to PC2 variation. TOC and TC explain 96.94% and 91.09% of the variation in microbial community changes, respectively.

### 3.8 Soil environmental impact effect

**Figure 11:**
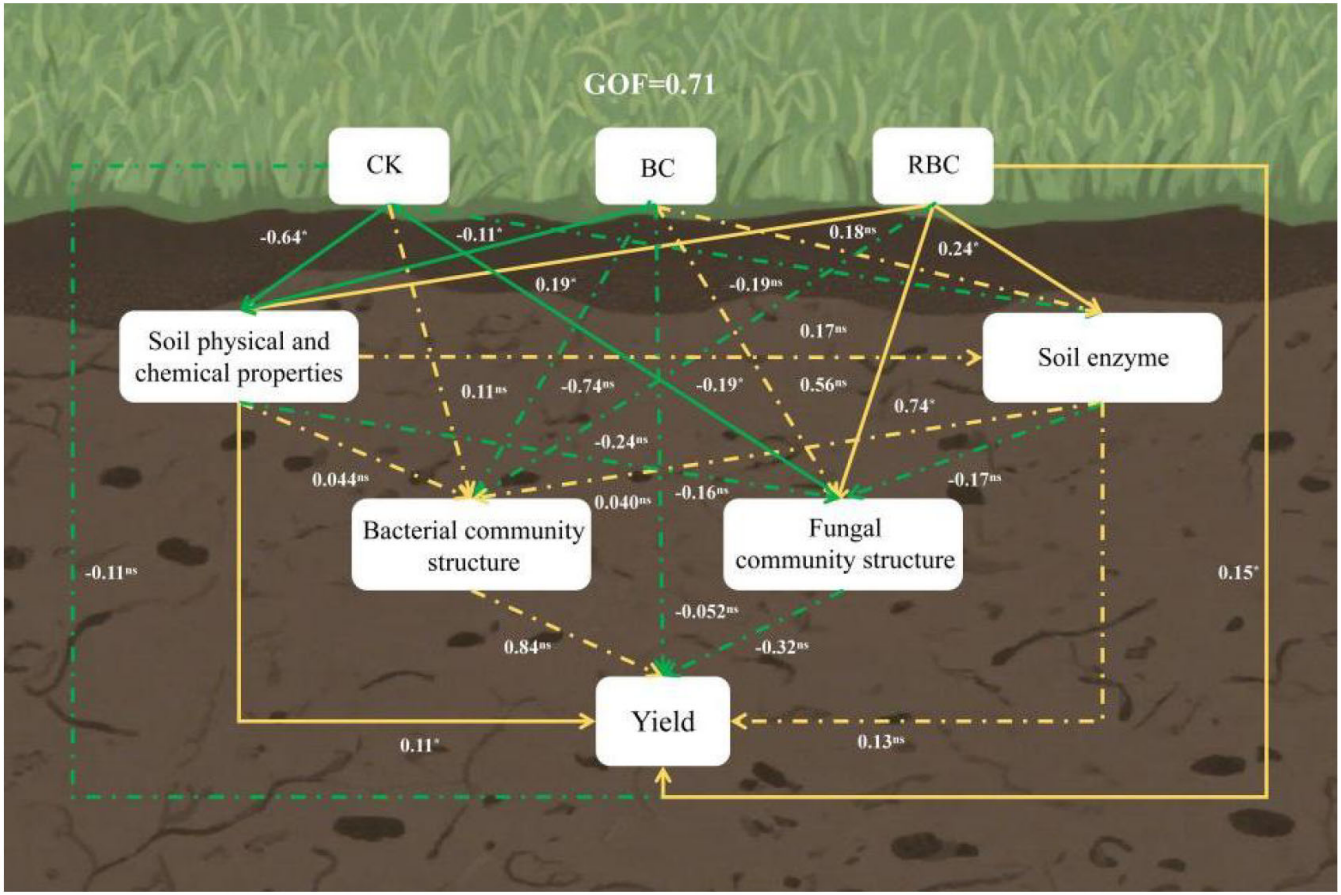
Partial least squares path modeling analysis. GOF represents the goodness of fit of the PLS-PM model. The numbers next to the lines represent the standardized path coefficients (i.e., direct effects). Solid lines indicate significant direct effects, while dashed lines indicate non-significant direct effects. “*” denotes significant direct effects (P < 0.05), and “ns” denotes non-significant direct effects (P > 0.05). Green lines indicate negative direct effects, and yellow lines indicate positive direct effects.

Partial Least Squares Path Modeling (PLS-PM) was used to further elucidate the potential relationships of modified biochar application on soil physicochemical properties, soil enzyme activity, soil microorganisms, and crop yield (**Figure 11**). The model’s goodness of fit was 0.71, indicating that the model’s outcomes adequately represent the interrelationships among the variables. The CK treatment had a certain degree of negative effects on soil physicochemical properties, enzyme activity, fungi, and yield. The application of biochar (BC) reduced the negative effects on soil physicochemical properties and yield, while also promoting positive effects on soil enzyme activity and fungi, but it exacerbated the negative impact on soil bacteria. The RBC treatment significantly enhanced the positive effects on soil physicochemical properties, enzyme activity, and fungi. Additionally, the RBC treatment alleviated the negative effects on soil bacteria compared to BC. Furthermore, soil physicochemical properties had a positive effect on soil bacteria, a negative effect on soil fungi, and a significant positive effect on yield.

## 4 Discussion

### 4.1 Effects of rhamnolipid-modified biochar on soil physicochemical properties

Biochar is an organic, carbon-rich material with high stability, which regulates the soil nitrogen and carbon cycles, as well as physicochemical properties, through multiple pathways (Pan et al., 2021). This study found that the application of biochar improves soil physicochemical properties, while rhamnolipid-modified biochar (RBC) significantly outperformed BC in increasing soil total nitrogen, total carbon, and organic carbon by 30.33% – 45.20%, 28.66% – 55.76%, and 244.07% – 370.10%, respectively. This increase does not represent soil carbon stabilization; rather, the application of exogenous modified biochar acts as a functional scaffold for soil carbon content. Unlike native organic matter, the stable porous structure provided by modified biochar serves as a physical carrier for the adsorption and retention of carbon and nitrogen (Lehmann et al., 2011). Additionally, Rhamnolipid enhances the dispersibility of biochar through emulsification, improving its adsorption and retention capacity for soil organic matter (Li et al., 2023). The RBC treatment reduced total dissolved solids by 39.40% – 56.78% and increased moisture content by 25.61% – 42.42%, demonstrating greater effectiveness than unmodified biochar. This indicates a synergistic water retention mechanism between rhamnolipid and biochar. This may be due to the presence of both hydrophilic and hydrophobic groups in the rhamnolipid molecules, which, through their strong chelation ability, effectively bind with soil base cations. Additionally, the carboxyl groups in rhamnolipid can form insoluble complexes with Na^+^, reducing soil colloid adsorption of Na^+^ (Berto et al., 2016; Zeng et al., 2018) Furthermore, rhamnolipid enhances the wettability of soil colloids, further optimizing soil pore structure and reducing moisture evaporation and leakage (Liu et al., 2018).

Soil enzymes play a crucial role in soil nutrient cycling and metabolism (Li et al., 2024). This study found that the application of rhamnolipid-modified biochar (RBC) significantly enhanced the activities of urease, sucrase, and cellulase, all of which were higher than those observed in the BC treatment. This increase is directly related to soil carbon and nitrogen content. Urease participates in soil nitrogen mineralization, and its increased activity accelerates the transformation of organic nitrogen into inorganic nitrogen, thereby supplementing the nitrogen requirements of peanuts (Kumar et al., 2024). Sucrase and cellulase are involved in carbon source decomposition. Rhamnolipid-modified biochar, by increasing the input of organic carbon, provides abundant substrates for subsequent microbial proliferation and indirectly activates enzymes involved in the carbon cycle (Bhatia et al., 2024). The alkaline phosphatase activity in the RBC treatment was lower than in the BC treatment but higher than in the CK treatment. This could be due to rhamnolipid, through emulsification, converting long-chain organic phosphates into shorter-chain molecules, thus reducing the substrate available for enzyme action (Raj et al., 2021). As an antioxidant enzyme, the increased activity of catalase helps alleviate oxidative damage under salt stress (Karim et al., 2025). The findings indicated that after the application of biochar, catalase activity in the soil decreased, with RBC treatment showing significantly higher activity than the BC treatment. This suggests that the application of biochar reduces soil salinity, decreases oxidative stress, and stabilizes the need for catalase. However, rhamnolipid-modified biochar, with its rich array of oxygen-containing functional groups and more oxygenated compounds, may have triggered oxidative stress in the soil, promoting an increase in catalase activity (Jing et al., 2026). In conclusion, the synergistic effect of rhamnolipid and biochar optimizes soil physicochemical properties, creating a suitable environment for soil enzyme activity and enhancing soil nutrient conversion efficiency.

### 4.2 Effects of rhamnolipid-modified biochar on microbial communities

Soil microbial diversity is a key indicator of soil ecological functions, and the application of biochar can directly or indirectly alter the soil microenvironment to influence soil bacterial abundance (Bamminger et al., 2016; Dai et al., 2021). The study found that, under saline conditions, the soil retained more salt-tolerant bacterial species, which formed specific community structures under salt stress and maintained the microbial ecological functions observed in the CK treatment. After biochar application, the bacterial Observed species, Ace, and Chao1 indices decreased. This could be attributed to the addition of biochar, which has high porosity and a large specific surface area. Its surface functional groups exhibit a strong affinity for nutrients, reducing the availability of nutrients directly accessible to bacteria in the soil. As a result, this restricts the growth and proliferation of most species within the bacterial community, leading to a decrease in bacterial alpha diversity and abundance (Wang et al., 2024). However, the reduction in RBC treatment was smaller than that in BC treatment, indicating that rhamnolipid modification alleviated the limitation of single biochar on bacterial diversity. Additionally, the reduction in total dissolved solids and the enhancement of nutrient capacity provided a more stable living environment for bacteria. In the fungal community, the use of rhamnolipid-modified biochar increased the Observed species, Ace, and Chao1 indices, with greater increases than the BC treatment. This suggests that fungi are more selective for the quality and form of carbon sources, particularly relying on small-molecule organic carbon as a metabolic substrate (Pawłowska et al., 2019; Sabuda et al., 2022). Although the application of biochar constructs a highly stable exogenous carbon pool, its dense structure results in slow organic carbon release, primarily in the form of complex macromolecules, making it difficult for fungi to efficiently utilize (Zhang et al., 2025). However, due to the improved dispersibility of rhamnolipid-modified biochar, it releases small molecules of organic matter more readily. This efficient carbon source provides abundant metabolic substrates for fungi, promoting the proliferation of carbon-dependent microbial groups such as *Ascomycota* (increased by 15.01%).

Soil microbial diversity serves as a key indicator of soil ecological functions, and the application of biochar can directly or indirectly alter the soil microenvironment, influencing soil bacterial abundance (Bamminger et al., 2016; Dai et al., 2021). The study found that in the bacterial community, *Proteobacteria*, *Firmicutes*, *Planctomycetes*, and *Acidobacteria* were the dominant phyla, consistent with studies on biochar application in saline soils (Wang et al., 2024). Interestingly, the application of rhamnolipid-modified biochar enriched the relative abundance of *Proteobacteria* (+1.63%), *Firmicutes* (+3.35%), and *Acidobacteria* (+17.39%), with the increase in *Firmicutes* being smaller than that in the BC treatment. This may be because *Proteobacteria*, as a eutrophic group, showed a positive correlation with higher soil urease activity and total nitrogen content in the RBC treatment, promoting nitrogen conversion efficiency (Li et al., 2019). *Firmicutes* are key contributors to soil carbon and nitrogen storage and nutrient cycling. Their unique cell wall structure gives them a specific preference for carbon source types, making it easier to utilize directly inputted, difficult-to-degrade carbon sources (SChade and Weidenmaier, 2016; Zhang et al., 2023a). In contrast, the BC treatment, with its porous structure, adsorbs and retains more macromolecular organic carbon, providing *Firmicutes* with richer exclusive substrates, leading to a higher increase in abundance. *Acidobacteria* are more sensitive to carbon sources, depending on high organic carbon environments, and their abundance exhibits a negative correlation with soil salinity (De Chaves et al., 2019; Li et al., 2021). The increase in soil organic carbon and the decrease in total dissolved solids in the RBC treatment further explain the increase in *Acidobacteria* abundance. At the genus level, *Neobacillus* and *Luteitalea*, salt-tolerant functional bacteria, showed increased abundance after the application of rhamnolipid-modified biochar (Ejaz et al., 2023), indicating that rhamnolipid-modified biochar promoted plant growth and enhanced salt tolerance. In the fungal community, the application of biochar increased the abundance of *Ascomycota*, with the RBC treatment showing a 15.01% increase in *Ascomycota* abundance. *Ascomycota*, as salt-tolerant fungi, secrete cellulase, ligninase, and other enzymes to degrade complex organic matter (Arfi et al., 2013). The increase in Ascomycota abundance is directly related to the increase in organic carbon and the decrease in salt content in the RBC treatment, while the BC treatment, with a lower increase in organic carbon and higher salt residue, limited its proliferation. *Chytridiomycota*, a characteristic group of moist soils (Blaalid and Khomich, 2021), had an abundance of 2.17% in the CK treatment. After the addition of modified biochar, soil moisture content increased by 25.61%-42.42%, leading to a significant increase in its abundance by 76.30%, confirming the optimization of soil wettability. Zoopagomycota are predominantly parasitic fungi, which can inhibit pathogenic fungi through nutrient competition or the secretion of antimicrobial substances (Arnesen et al., 2018). They were nearly undetectable (<0.001%) in the CK treatment, but their abundance increased after biochar application, with the RBC treatment resulting in a 755.29% increase, indicating that RBC stimulated the proliferation of these fungi and indirectly promoted soil health. Basidiomycota primarily exist in a saprophytic form, decomposing limited organic residues. In stressed saline-alkali soil ecosystems, the RBC treatment led to a decrease in their abundance, which may be beneficial for the health of plant seedlings. At the genus level, the RBC treatment drove an increase in the abundance of *Aspergillus*, *Penicillium*, and *Fusarium*. *Aspergillus* and *Penicillium*, typical plant-promoting fungi, secrete auxins and extracellular enzymes (Imran et al., 2021; Mukherjee et al., 2023). Their enrichment, together with high cellulase activity in the RBC treatment, accelerates carbon source conversion. Some strains of *Fusarium* participate in cellulose degradation, which matches the abundant organic carbon supply in the RBC treatment (Najjarzadeh et al., 2021).

Species interactions within microbial communities mirror organic matter decomposition, the exchange of organic carbon substances, and microbial community functions (Banerjee et al., 2016). Highly connected community networks further enhance nutrient decomposition efficiency, increase functional redundancy, and promote the distributed expression of environmental resistance (Du et al., 2022). This study found that in saline soils, bacterial communities maintain ecological balance through diversified interspecific competition and cooperation, and the modularity degree reflects the community’s adaptive differentiation to stress environments. The BC treatment reduced the connectivity of the bacterial community, which may be due to biochar containing recalcitrant carbon sources that lower soil mineralization, thus being detrimental to microbial interactions (Yu et al., 2022). In the modified biochar network, the number of positive correlations between core bacterial phyla significantly increased, functional redundancy gradually enhanced, and the interspecies cooperation ability of biochar was strengthened. This improvement is due to rhamnolipid enhancing the pore structure of biochar (Jing et al., 2026), allowing more bacteria to coexist and increasing the community’s resilience to environmental fluctuations. In the fungal co-occurrence network, the CK treatment, due to high salinity and low carbon environments, limited fungal diversity, with only a few salt-tolerant strains forming weak associations and poor functional synergy (Zhang et al., 2023b). However, the incorporation of biochar optimized the fungal microecology in the soil, and the application of rhamnolipid-modified biochar significantly enhanced the synergistic interactions within the community. Core fungal phyla such as *Ascomycota* and *Chytridiomycota* formed more stable functional modules through positive correlations, which were directly related to the reduction in salt content and activation of carbon sources in the RBC treatment. The increased modularity of the network further promoted community stability. These results suggest, to some extent, that rhamnolipid-modified biochar regulates the soil microenvironment, alleviating the decline in bacterial diversity, enhancing fungal richness, enriching beneficial functional microbial groups, and inhibiting harmful microorganisms, while also strengthening interspecies cooperation.

### 4.3 Synergistic effect of soil quality and environmental factors on yield

Soil microorganisms are highly sensitive to multiple environmental factors, including humidity, pH, temperature, and soil nutrients, all of which strongly influence the composition of microbial communities (Wu et al., 2022). In this study, the composition of bacterial and fungal populations exhibited significant correlations with soil physicochemical properties and soil enzyme activities. Within the bacterial community, TOC displayed a positive correlation with most bacterial phyla, while TC and SUC were positively correlated with *Chloroflexi* and *Actinobacteria*, respectively. These relationships arise because both groups are regulated by soil carbon sources, and higher soil carbon content is more conducive to their growth (De Chaves et al., 2019). In contrast, TDS showed a strong negative correlation with *Chloroflexi* and *Actinobacteria*. Although *Actinobacteria* are generally regarded as salt-tolerant, their growth is substantially inhibited under high salt stress (Xiong et al., 2019). Similarly, the overall salt tolerance of *Chloroflexi* at the phylum level is limited, with its relative abundance decreasing as soil salinity increases (Zhao et al., 2018). For the fungal community, *Ascomycota* exhibited a significant positive correlation with TC and TOC, reflecting its strong adaptability to organic carbon environments and its competitive advantage in resource utilization (Arfi et al., 2013). In contrast, both *Basidiomycota* and *Mucoromycota* displayed significant negative correlations with TC and TOC. *Basidiomycota* is typically a “specialized slow-growing” saprophytic fungus that thrives in environments with low nitrogen and low easily degradable carbon (Krupodorova et al., 2021), whereas *Mucoromycota* contributes to the decomposition of soil organic matter, with its abundance also showing a significant negative correlation with TN and TC (Fernandez and Kennedy, 2018). Additionally, Meanwhile, Zoopagomycota showed a significant positive correlation with peanut yield (Y), which may be due to the ensured absorption of water and nutrients by the peanut roots, thereby driving changes in peanut yield (Arnesen et al., 2018). Additionally, random forest analysis revealed that TC, TOC, and SCL are the most explanatory variables for predicting microbial community changes, with the largest weight. This indicates that the efficient activation of microbial functions plays an irreplaceable role in the conversion of TC and TOC and the carbon cycling process mediated by SCL.

The formation of yield in the dynamic process of crop growth and development is not the result of a single factor, but rather the combined effect of soil quality and environmental factors working synergistically (Adare et al., 2023; Pandey and Kamboj, 2022). In this study, the Soil Quality Index (SQI), which integrates multiple soil physicochemical indicators, was used to evaluate the comprehensive impacts of modified biochar application on soil physicochemical properties, enzyme activities, and microbial communities. The results demonstrated that biochar application significantly increased SQI, which was strongly and positively correlated with yield. Among the treatments, RBC produced the greatest yield increase, suggesting that rhamnolipid-modified biochar substantially improves soil quality and, consequently, peanut yield. Partial Least Squares Path Modeling (PLS-PM) was employed to analyze the path relationships among soil physicochemical properties, enzyme activity, and microbial populations across treatments. The CK treatment exhibited negative effects on soil physicochemical properties, enzyme activity, fungal communities, and yield, thereby reflecting the constraints imposed by saline soils. In contrast, the BC treatment alleviated some negative effects but simultaneously strengthened inhibition of the bacterial community. The RBC treatment, however, enhanced positive effects on soil physicochemical properties, stimulated enzyme activity, and regulated the fungal community, while mitigating bacterial inhibition, thereby establishing a synergistic network structure. Furthermore, soil physicochemical characteristics exerted a significant positive direct effect on yield, and, together with enzyme activity and microbial communities, formed a multidimensional support system for yield improvement.

## 5 Conclusion

Rhamnolipid-modified biochar alleviated salinized substrates and enhanced soil productivity in arid regions. Compared to single biochar, it significantly increased the total carbon stock in the 0–20 cm soil layer by triggering the chain regulatory mechanism of the “soil-microbe-crop” system and optimized the soil physicochemical environment related to nutrient transformation, thereby creating more favorable conditions for peanut growth. Moreover, modified biochar directed the regulation of soil microbial community structure, alleviated the limitation of single biochar on bacterial diversity, increased fungal diversity, and enhanced the synergistic effects of microbial co-occurrence networks, leading to improved soil quality and a 27.57% increase in peanut yield. This study reveals the potential pathways by which rhamnolipid-modified biochar enhances soil quality and peanut yield in saline-alkali soils of southern Xinjiang through physicochemical and microbial mechanisms. It also indirectly highlights the potential ecological risk of reduced soil bacterial communities associated with the application of biochar in saline-alkali soils.

Current research has demonstrated the superior performance of rhamnolipid-modified biochar; however, we also acknowledge that the application rate of 4 kg·m^-2^ represents a relatively high initial investment for farmers. This dose was selected primarily to maximize the observation of the “soil-microbe-crop” chain regulatory mechanism. Future research should explore the economically optimal dose for large-scale application under various saline-alkali environments, as well as the long-term cost-benefit ratio, to support the sustainable development of agriculture in saline-alkali soils.

## Data Availability

The datasets presented in this study can be found in online repositories. The names of the repository/repositories and accession number(s) can be found below: https://www.ncbi.nlm.nih.gov/, PRJNA1304183. The sequences of 16S rRNA and ITS rDNA have been deposited in the Sequence Read Archive (SRA) at the National Center for Biotechnology Information (NCBI), with the bioproject accession number PRJNA1304183 (biological sample accession numbers for bacteria from field and culture experiments are SRR34943096 - SRR34943102 and SRR34943111 - SRR34943112; for fungi, they are SRR34943095).
